# Effect of theta burst stimulation on lower extremity motor function improvement and balance recovery in patients with stroke: A systematic review and meta-analysis of randomized controlled trials

**DOI:** 10.1097/MD.0000000000040098

**Published:** 2024-11-01

**Authors:** Kang Chen, Meixia Sun, He Zhuang

**Affiliations:** a School of Rehabilitation Medicine, Shandong University of Traditional Chinese Medicine, Jinan, Shandong, China.

**Keywords:** balance, lower extremity motor function, meta-analysis, stroke, theta burst stimulation

## Abstract

**Background:**

To investigate the therapeutic benefits of theta burst stimulation on lower-limb motor dysfunction and balance recovery in patients with stroke.

**Methods:**

A literature search was performed across CNKI, CBM, WanFang, VIP, PubMed, Embase, Cochrane Library, and Web of Science until November 2023. The Methodological quality of included studies was assessed by using the Cochrane risk-of-bias tool and the PEDro scale, and the meta-analysis was performed by using RevMan 5.3 software. Two independent researchers screened the literature and extracted basic information on participants, interventions, comparisons, outcomes, and studies.

**Results:**

Eight studies, including cTBS and iTBS, with 290 participants meeting the inclusion criteria for this systematic review, and 7 studies including only iTBS with 230 participants were included in this meta-analysis. The methodological quality of the studies included ranged from moderate to high. The results showed iTBS had significantly higher scores on the Berg Balance Scale (BBS) than the control group. (MD = 4.57, 95% CI: 1.76 to 7.38, *Z* = 3.19, *P* = .001). Subgroup analysis showed CRB-iTBS markedly improved BBS scores (MD = 4.52, 95% CI: 1.78 to 7.27, *Z* = 3.23, *P* = .001), whereas LE M1-iTBS did not exhibit a significant enhancement in BBS scores (MD = 6.10, 95% CI: −7.34 to 19.53, *Z* = 0.89, *P* = .37); iTBS showed no significant increase in lower-limb motor function (FMA-LE) (MD = 1.80, 95% CI: −1.10 to 4.69, *Z* = 1.22, *P* = .22). Subgroup analysis revealed both CRB-iTBS and LE M1-iTBS interventions were not effective in improving FMA-LE (MD = 3.15, 95% CI: −4.70 to 11.00, *Z* = .79, *P* = .43; MD = 1.05, 95% CI: −2.20 to 4.30, *Z* = .63, *P* = .53); iTBS significantly reduced the MEP latency (*P* = .004), but did not show a significant improvement in walking performance (10 MWT), mobility (TUG), or activities of daily living [M(BI)] (*P* > .05).

**Conclusion:**

Based the current study, iTBS can increase patients’ balance function. The CRB-iTBS protocol is more effective than the LE M1-iTBS protocol. Additionally, iTBS may be a promising therapy tending to enhance lower-limb motor function, walking performance, mobility, and activities of daily living.

## 1. Introduction

Stroke is a common cerebrovascular accident that includes both hemorrhagic and ischemic types, resulting in substantial medical and societal burden on a global scale.^[1]^ The prevalence of stroke in China is reported to be on the rise, reaching as high as 2.6% among individuals aged 44 years and older.^[2]^ Stroke is the primary cause of death and long-term disability among Chinese residents.^[3]^ In addition, individuals with hemiplegia resulting from a stroke tend to have motor impairment in their limbs, particularly in the lower-limb. Approximately 88% of patients experience lower-limb motor dysfunction, resulting in a loss of mobility, walking difficulties, and a greater susceptibility to falls.^[4,5]^ The presence of lower-limb motor dysfunction and balance impairment significantly restricts patients’ ability to be independent, limits their social participation, and reduces their overall quality of life. Therefore, a crucial goal for stroke patients is to improve lower-limb motor function, particularly walking capacity, mobility, and balancing function recovery.^[6]^

At present, the primary therapeutic methods for enhancing motor and balance capabilities in the lower limbs include manual therapy, gait training, and the use of lower-limb exoskeleton robots and dynamic balance instruments.^[6,7]^ Nevertheless, the therapy options currently accessible are limited, and the effectiveness of improving lower-limb motor function and balance recovery varies among patients. In recent years, repetitive transcranial magnetic stimulation (rTMS) has emerged as a promising noninvasive brain stimulation technique for patients with lower-limb dysfunction after stroke.^[8]^ This provides a novel method for increasing lower-limb motor function and regulating corticospinal excitability, which controls muscle movements in the bilateral lower limb. In particular, rTMS uses theta burst stimulation (TBS) as a patterned paradigm. TBS differs from rTMS in that it involves high-frequency pulses, low stimulation intensity, and a short stimulation time (80–300 seconds). Nevertheless, TBS produces long-term potentiation of the synaptic strength in the cerebral cortex, resulting in a duration of 20 to 30 minutes, which is similar to that of rTMS.^[9,10]^

Regarding the primary mechanism of TBS, TBS works by simulating and releasing external pulses at 5 Hz, a frequency that resembles the theta frequency observed in the motor cortex and hippocampal region during motor learning and body schema updating. This process facilitates changes in the excitability and plasticity enhancement of the cerebral motor cortex, promoting poststroke recovery.^[11]^ In addition, TBS can upregulate the levels of cerebral vascular protective and neurotrophic factors, hence promoting the process of recovery after stroke injury.^[12,13]^ Notably, there are 2 different types of stimulation in TBS: intermittent theta burst stimulation (iTBS) and continuous theta burst stimulation (cTBS). TBS primarily produce either excitatory or inhibitory effects by stimulating various regions. Specifically, iTBS primarily targets the affected side of the primary motor cortex (M1) and the contralateral cerebellum (CRB) region to generate cortical long-term potentiation effects and enhance excitability. On the other hand, cTBS mainly activates the contralateral M1 region to induce long-term depression effects and contribute to the suppression of cortical excitability.^[9,10]^

Currently, TBS is being researched as a potential noninvasive brain stimulation technique for improving lower-limb functional impairments and promoting balance recovery in patients with stroke. While some studies have shown promising results, there are inconsistent reports regarding the efficacy of TBS in enhancing motor impairments and balance function in the lower limbs following stroke. For instance, 1 previous study^[14]^ reported significant improvement in lower-limb motor function and balance recovery in stroke patients, while Koch et al^[15]^ found that iTBS can contribute to the restoration of balance, but its clinical effectiveness in enhancing limb motor function is not significant. Furthermore, there is currently a lack of recommended treatment parameters and evidence-based medicine for the use of TBS in lower-limb rehabilitation after stroke. Therefore, in this review, we aimed to systematically evaluate the intervention effects, trial protocols, and optimal treatment parameters of TBS in improving lower-limb motor impairments and balance recovery in stroke patients.

## 2. Material and methods

### 2.1. Protocol and registration

The Preferred Reporting Items for Systematic Reviews and Meta-Analyses (PRISMA) guidelines were followed in performing this systematic review and meta-analysis.^[16]^ The protocol has been registered at the International Prospective Register of Systematic Reviews (PROSPERO, No.CRD42023487673).

### 2.2. Literature search and strategy

The following databases were searched from inception to November 2023: CNKI, CBM, WanFang, VIP, PubMed, Embase, Cochrane Library and Web of Science. In addition, we performed a manual search from TBS review and relevant references of randomized controlled trials (RCTs) included in this meta-analysis. The retrieval strategy was developed and performed using a combination of Mesh terms and free words, and restricted to publications in Chinese or English.

The following Chinese and English search terms were used: #1 (disease): stroke, cerebrovascular accident, cerebrovascular apoplexy, brain vascular accident, cerebrovascular stroke, apoplexy, cerebral stroke, acute stroke, acute cerebrovascular accident, and chronic stroke; #2 (function or dysfunction): motor, movement, motion, mobility, movement function, motor function, motor dysfunction, motor impairment, movement dysfunction, movement impairment, lower extremity, lower-limb, balance, gait, and walking; and #3 (intervention): TBS, cTBS, and iTBS.

### 2.3. Inclusion and exclusion criteria

Inclusion criteria were reports of RCTs that focused on the effects of TBS on lower-limb motor function and balance in patients with stroke. Studies were selected for inclusion based on the PICOS (population, intervention, compare, outcomes, study design) criteria. Table 1 presents a detailed list of inclusion criteria.

**Table 1:** The inclusion criteria for literature based on the PICOS principle.

PICOS principle	Inclusion criteria
Population	(1) The patient must have received a diagnosis of stroke, including hemorrhagic or ischemic stroke, through cranial imaging, such as MRI or CT; (2) patients suffer from motor or balance dysfunction in the lower limbs; (3) patient’s age should be over 18 years (average age) and condition should be in the subacute or chronic phase; (4) there are no any limitations on gender, race, religious beliefs, or nationality.
Intervention	TBS (iTBS or cTBS) or TBS combined with other interventions therapy.
Comparison	Sham TBS or Sham TBS combined with other intervention therapy.
Outcomes	(1) The primary outcome:the Fugl-Meyer Assessment of Motor Recovery for lower extremity (FMA-LE), the Berg Balance Scale (BBS); (2) the secondary outcome:the (modified) Barthel Index[(M)]BI, 10-m walk test (10MWT), the Timed UP and GO(TUG), cortical spinal excitability assessments such as motor evoked potentials (MEP); (3) to be eligible for inclusion, the meta-analysis must incorporate the FMA-LE or BBS as part of the outcome measures.
Study design	RCTs.

cTBS = continuous theta burst stimulation, FMA-LE = Fugl-Meyer assessment of motor recovery for lower extremity, iTBS = intermittent theta burst stimulation, RCTs = randomized controlled trials, TBS = theta burst stimulation.

In addition, the exclusion criteria applied in this meta-analysis were as follows: duplicate publications; systematic reviews, meta-analyses, conference papers, expert comments, animal experiments, and master’s or doctoral theses; cross-sectional studies, case–control studies, cohort studies, other types of observational studies, case reports, or non-RCTs; literature with significant bias, such as baseline imbalances, conflicts of interest, or small sample sizes (n < 8); literature with inconsistent study designs, interventions, control measures, outcome indicators, or without data on primary outcome indicators; literature for which full texts were inaccessible; and patients in the acute phase.

### 2.4. Literature screening and data extraction

Two researchers (Chen K and Sun M) employed NoteExpress 3.7.0 independently to manage the literature. Initially, we screened the literature, removing duplicates and ineligible studies based on titles and abstracts. Subsequently, we identified the literature depending on the full texts, and ultimately selected literature that met the eligibility criteria. Any disagreements have been settled by discussion with a third researcher (Zhuang H).

The data extraction process involved the following aspects: basic characteristics, including the first author, publication year, age, disease progression, sample size, interventions, outcomes, and adverse reactions; TBS parameters, such as the treatment device, stimulation area and mode, site and intensity of stimulation, overall duration of stimulation, total number of pulses, and treatment duration; and methodological information, including randomization, allocation concealment, and blinding. To obtain missing data, we contacted with the original authors by email.

### 2.5. Methodological assessment

#### 2.5.1. Bias risk assessment of included studies

Two autonomous reviewers (Chen K and Sun M) performed a qualitative evaluation using the Cochrane risk-of-bias instrument (RoB) to detect potential bias and assess internal validity in RCTs.^[17]^ The assessment process included following essential areas. For selection bias, the evaluation focused on the execution of randomized sequence generation and allocation concealment. This involved assessing whether the study employed suitable randomized procedures to create sequences and whether allocation concealment prevented selective grouping. Subsequently, implementation bias was examined, specifically on the execution of blinding for both subjects and researchers. Blinding was implemented to ensure that both the subjects and researchers were unaware of the treatment allocation, thereby minimizing the impact of expectation effects. For assessment bias, the evaluation of blinding in outcomes was conducted to minimize subjective bias. For follow-up bias, the completeness of outcome data was assessed, including analysis of missing data and researchers withdrawals from the study. In addition, the study was evaluated for reporting bias, which entailed examining whether the study reported all prespecified outcomes and whether there was selective reporting that could affect intervention effects. Finally, other biases were assessed, including baseline comparability and conflicts of interest. Based on the above assessments, the risk-of-bias was categorized as high, low, or unclear.

#### 2.5.2. Literature quality assessment of included studies

The quality of the included studies was assessed using the PEDro scores obtained from the Physiotherapy Evidence Database. The PEDro scale contains 11 items, with each item contributing 1 point to the overall score (ranging from 0 to 10), except for the first item, which is not assigned a score. Quality scores range from 6 to 10 for high quality, 4 to 5 for moderate quality, and 0 to 3 for low quality.^[18]^ The assessment process involved evaluating the methods of randomization and allocation; subsequently, an assessment was conducted to determine whether the baseline data for the 2 groups were comparable; additionally, blind evaluation was performed for the participants, therapists, and assessors; furthermore, the extent to which data were reported in full for 85% of participants was evaluated, and missing data whether was addressed using an intention-to-treat analysis; finally, point estimates and variability was also examined.

#### 2.5.3. Publication bias analysis

The analysis of publication bias was not conducted in this study, as the necessary number of studies or data points for a valid assessment was not available.

### 2.6. Statistical analysis

Between-group meta-analysis was performed using RevMan 5.3 software (Cochrane, London, UK). The baseline of the intervention and control groups in the included studies was comparable, thus the outcome data at the conclusion of the treatment period were extracted. As this meta-analysis focused on continuous outcomes, the means and standard deviations were extracted. Given that the scales or units of measurement used in all studies were consistent, the combined effect sizes and the related variability of continuous outcomes were presented as mean difference (MD) and 95% confidence intervals (95% CI).

The evaluation of statistical heterogeneity was performed using the *I*^2^ statistic and its associated *P* value. The values were classified into 3 levels based on their heterogeneity: low (*I*^2^ ≤ 50% and *P* ≥ .1), moderate (50% <  *I*^2^ < 75% and *P* < .1), and high (*I*^2^ ≥ 75% and *P* < .1).^[19]^ To account for the varying levels of heterogeneity, a fixed-effects model was used for the pooled data with low heterogeneity, while a random-effects model was used for the pooled data with moderate to high heterogeneity. A sensitivity analysis was performed for alleviating the statistical heterogeneity.

To assess the impact of various TBS trial protocols (M1 LE-iTBS vs CRB-iTBS) on the overall effect sizes, we conducted subgroup analyses for the primary outcomes (FMA-LE and Berg Balance Scale [BBS] scores). All statistical significance levels have been set as *P* < .05.

## 3. Results

### 3.1. Literature search results and research selection

**Figure 1.**
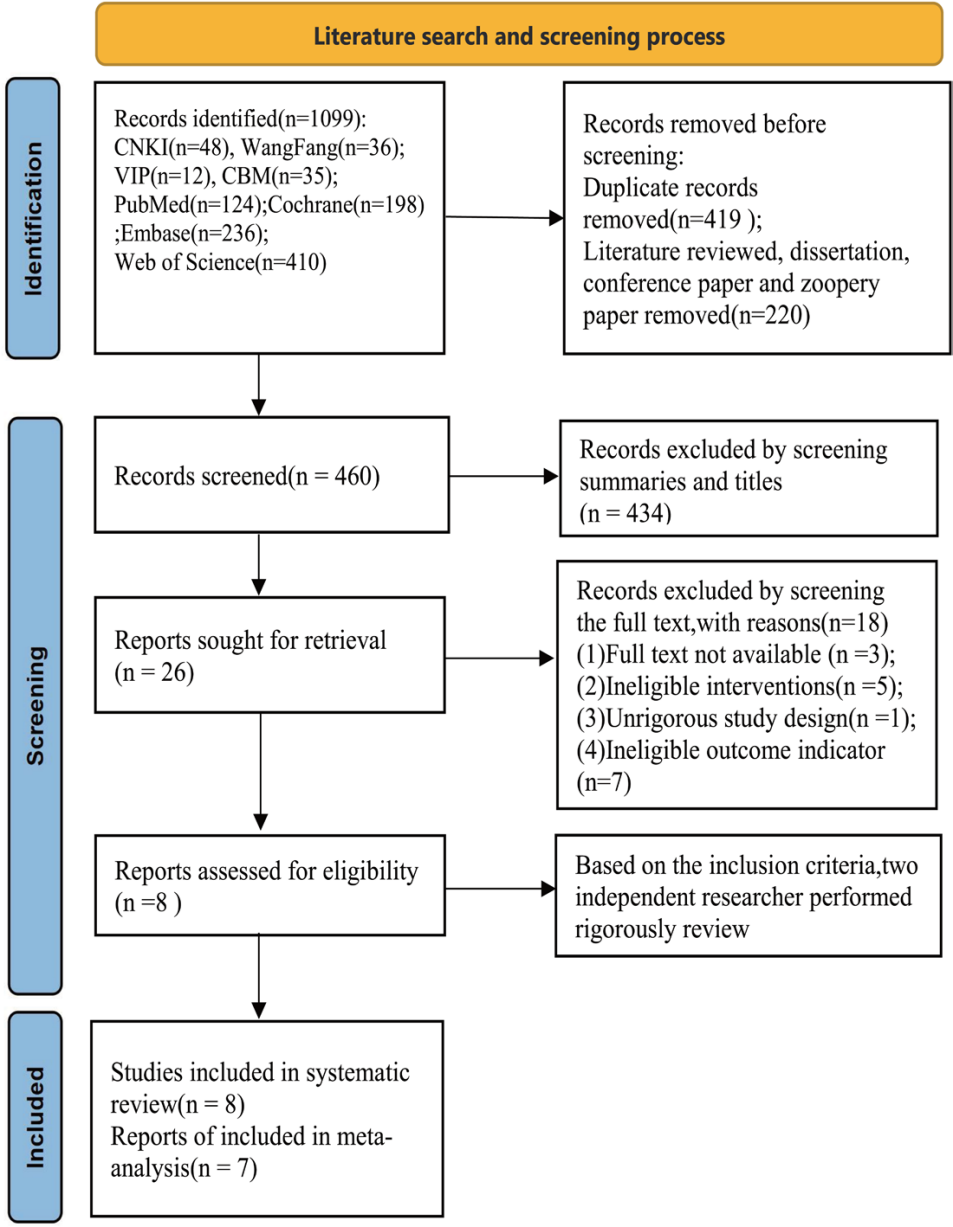
Flow chart for screening included studies.

Figure 1 shows a detailed illustration of the process of screening the literature. A total of 1099 articles were retrieved from databases previously mentioned above. After removing duplicate articles and excluding reviews, meta-analysis, conference papers, theses, animal studies, and so on, there were 460 articles remaining. Afterwards, the process of screening the titles and abstracts led to the identification of 26 articles. Following a full-text review, a total of 8 studies^[14,15,20–25]^ were found to meet the criteria for a systematic review. Of the 8 eligible studies, a total of 7 studies^[14,15,20–23,25]^ were included in the meta-analysis, with 3 studies published in Chinese^[14,20,21]^ and 4 studies in English.^[15,22,23,25]^

### 3.2. Methodological assessment results

#### 3.2.1. Bias risk of included literature

**Figure 2.**
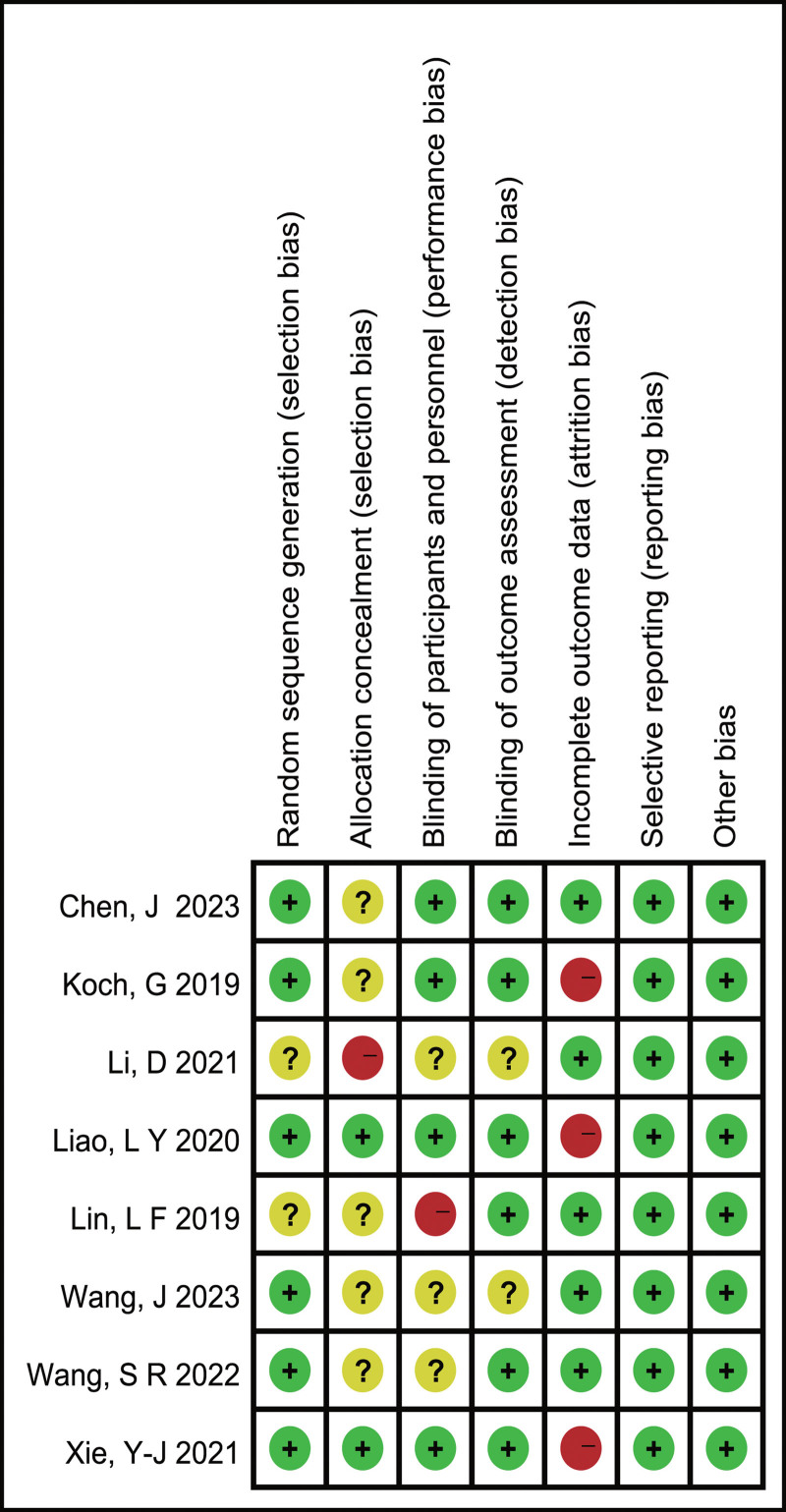
Bias risk of included systematic review in literature. “+”:low bias risk; “?”:unclear risk; “-”:high bias risk.

Figure 2 shows a detailed summary of bias risk in the included studies. All studies utilized randomization. Among them, Four studies^[14,20,21,23]^ used random number generation, 1 study^[22]^ used computer-generated random sequences, and 1 study^[15]^ used the minimal sufficient balance method. However, the specific randomization method was not reported in other 2 studies.^[24,25]^ Regarding allocation concealment, 2 studies^[22,23]^ employed sealed and opaque envelopes to ensure concealment, 1^[24]^ had an open-label design, whereas allocation concealment in the remaining studies^[14,15,20,21,25]^ was not clearly defined. For double-blinding of both the researchers and participants, 3 studies^[15,21,22]^ used double-blinding. In 1 particular study,^[23]^ blinding was implemented for participants and therapists who offered conventional rehabilitation treatment (CRT), but not for therapists implementing the TBS intervention, which was deemed to have a low risk after consideration. Additionally, 1 study^[25]^ did not use double-blinding, while the blinding approach was unclear in the remaining 3 studies.^[14,20,24]^ For blind of outcome assessors, blinding was adopted in 6 studies,^[14,15,20–23,25]^ but clear blinded was lacking in 2 studies.^[20,24]^ Finally, the endpoint data in 3 studies^[15,22,23]^ were found to be lacking in completeness, but all of these studies utilized intention-to-treat analyses, which were considered to have a low risk.

#### 3.2.2. Methodological quality of included studies

Table 2 presents a detailed summary of the PEDro quality scores for the included studies. The PEDro quality scores of the studies varied from 5 to 10 points, with a mean score of 7.25. Of the 8 included studies, 6 of the studies^[14,15,21–23,25]^ were considered to be of high quality (score > 6 points), whereas 2^[20,24]^ were of low quality (score ranging from 4 to 5 points). In addition, 3 of the studies^[15,22,23]^ performed intention-to-treat analyses. All the indicated studies had sufficient follow-up (>85%), between- group comparisons, point estimates, and variability. However, the sample size did not meet the threshold required by the Methods section (n < 50).

**Table 2:** The PEDro quality scores for the included literature.

Items	Wang^[14]^ 2022	Wang^[20]^ 2023	Chen^[21]^ 2023	Xie^[22]^ 2021	Liao^[23]^ 2020	Li^[24]^ 2021*	Koch ^[15]^ 2019	Lin^[25]^ 2019
Eligibility criteria	Y	Y	Y	Y	Y	Y	Y	Y
Random allocation	1	1	1	1	1	1	1	1
Concealed allocation	0	0	0	1	1	0	0	0
Baseline comparability	1	1	1	1	1	1	1	1
Blinded subjects	0	0	1	1	1	0	1	0
Blinded therapists	0	0	1	1	0	0	1	0
Blinded assessors	1	0	1	1	1	0	1	1
Adequate follow-up (>85%)	1	1	1	1	1	1	1	1
Intention-to-treat analysis	0	0	0	1	1	0	1	0
Between-group comparisons	1	1	1	1	1	1	1	1
Point estimates and variability	1	1	1	1	1	1	1	1
Total PEDro score	6	5	8	10	9	5	9	6
Level of quality	H	M	H	H	H	M	H	H
Sample size ≥ 50	N	N	N	N	N	Y	N	N

H = high quality (6–10 points), M = moderate quality (4–5 points).

* Due to the outcome index data could not be extracted, this study was not included in the meta-analysis.

### 3.3. Basic information and characteristics

#### 3.3.1. Participants

Table 3 presents the characteristics of the included studies, and Table 4 includes the essential parameters of the TBS trial protocol. This systematic review included a total of 290 participants from 7 studies conducted in China^[14,20–25]^ and 1 study conducted in Italy.^[15]^ The age of the participants varied from 50 to 65 years, with a higher number of males (n = 189) compared to females (n = 101). Both hemorrhagic and ischemic stroke types were observed in all the studies. Of the 8 studies, 6^[14,20–24]^ included participants in the subacute stage of the disease, whereas the remaining 2 studies^[15,25]^ included participants in the chronic stage.

**Table 3:** The basic information and characteristics for included study.

Study	Design	Country	Age (yr)		Sample (male and female)		I/H	Disease duration		Intervention		Follow-up	Outcomes
			T	C	T	C		T	C	T	C		
Wang^[14]^ 2022	RCT	China	52.62 ± 8.61	54.62 ± 7.85	21 (11/10)	21 (9/12)	25/17	82.33 ± 45.27d	72.95 ± 47.37d	CRB-iTBS + CRT + suspension exercise	CRT + suspension exercise	No	
Wang^[20]^ 2023	RCT	China	54.83 ± 12.57	53.44 ± 16.60	18 (13/5)	18 (12/6)	22/14	6.61 ± 1.69w	7.12 ± 2.26w	LE M1-iTBS + CRT	Sham iTBS + CRT	No	
Chen^[21]^ 2023	RCT	China	58.88 ± 15.97	62.38 ± 12.66	16 (11/5)	16 (9/7)	29/3	3.75 ± 2.84m	3.88 ± 2.53m	CRB-iTBS + CRT	Sham iTBS + CRT	3w	
Xie^[22]^ 2021	RCT	China	52.35 ± 8.62	54.41 ± 7.01	18 (13/5)	18 (11/7)	20/16	2.22 ± 1.70m	2.91 ± 1.96m	CRB-iTBS + CRT	Sham iTBS + CRT	No	
Liao^[23]^ 2020	RCT	China	51.34 ± 9.22	55.40 ± 8.10	15 (12/3)	15 (9/6)	15/15	70.40 ± 44.34d	86.53 ± 45.26d	CRB-iTBS + CRT	Sham iTBS + CRT	No	
Li^[24]^ 2021*	RCT	China	56.77 ± 8.58	57.60 ± 7.40	30 (20/10)	30 (19/11)	47/13	3.63 ± 1.85m	3.80 ± 1.71m	CRB-cTBS + LF-rTMS + CRT + acupuncture	LF-rTMS + CRT + acupuncture	No	
Koch^[15]^ 2019†	RCT	Italy	63.00 ± 11.00	65.00 ± 12.00	17 (13/4)	17 (10/7)	-	7 (9.0) m	7 (3.5) m	CRB-iTBS + CRT	Sham iTBS + CRT	3w	
Lin^[25]^ 2019	RCT	China	60.81 ± 8.1	61.1 ± 9.7	10 (9/1)	10 (8/2)	16/4	359 ± 171 d	384 ± 270 d	LE M1-iTBS + CRT	Sham iTBS + CRT	No	

(M)BI = (modified) Barthel Index, 10MWT(s) = 10-m walk test(s), BBS = Berg Balance Scale, C = Control group, CRB-cTBS = cerebellar stimulation of cTBS, CRB-iTBS = cerebellar stimulation of iTBS, CRT = conventional rehabilitation treatment, FMA-LE = Fugl-Meyer Assessment of Motor Recovery for lower extremity, H = hemorrhagic, I = ischemic, LE M1-iTBS = iTBS stimulates M1 of low limb, MAS = modified Ashworth scale, MEP = Motor Evoked Potentials, POMA-G = Performance Oriented Mobility Assessment-Gait, sham iTBS = sham stimulation of iTBS, T = intervention group, TIS = the Trunk Impairment Scale, TUG = Timed Up and Go test; TMS-EEG test index.

* Due to the outcome index data could not be extracted, this study was not included in the meta-analysis.

† The duration of the disease is indicated by M (IQR).

**Table 4:** The essential characteristics for TBS parameters.

Study ID	Device and coil type	Stimulation site	Stimulation mode and intensity	Stimulation parameters	Total time (s)	Total pulses (n)	Treatment cycle	Side effect
Wang^[14]^ 2022	CCY-I; Figure-of-8 coil; 70-mm diameter	Contralateral cerebellum	CRB- iTBS; 80%AMT	Bursts of 3 pulses at 50 Hz applied at a rate of 5 Hz, a 2-s stimulation time with 8-s intervals	180	600	1 time/d, 180 s/time, 5 d/wk, lasting 4 wk	NO
Wang^[20]^ 2023	CCY-I; Conical coil	Affected LE M1	LE M1-iTBS; 70% RMT	Bursts of 3 pulses at 35 Hz applied at a rate of 5 Hz, a 2-s stimulation time with 8-s intervals, repeat 40 times	400s	1200	1 time/d, 400 s/time, 6 d/wk, lasting 3wk	NO
Chen^[21]^ 2023	CCY-I; Figure-of-8 coil	Contralateral cerebellum	CRB- iTBS; 80% RMT	Bursts of 3 pulses at 50 Hz applied at a rate of 5 Hz, a 2-s stimulation time with 8-s intervals	200s	600	1 time/d, 200 s/time, 6 d/wk, lasting 3 wk	NO
Xie^[22]^ 2021	CCY-I; Figure-of-8 coil; 70-mm diameter	Contralateral cerebellum	CRB- iTBS; 80% AMT	Bursts of 3 pulses at 50 Hz applied at a rate of 5 Hz,20 trains of 10 bursts at 8-s intervals	180s	600	1 time/d, 180 s/time, a total of 10 times, lasting 2 wk	NO
Liao^[23]^ 2020	CCY-I; Figure-of-8 coil; 70-mm diameter	Contralateral cerebellum	CRB-iTBS; 80% AMT	–	–	600	1 time/d, a total of 10 times, lasting 2 wk	NO
Li^[24]^ 2021^a^	–	The right cerebellum	CRB-cTBS; 80% AMT	Bursts of 3 pulses at 50 Hz,100 stimuli, a 20-s stimulation time with no intervals	80 s	1200	1 time/d, 80 s/time, 6 d/wk, lasting 4 wk	NO
Koch^[15]^ 2019	MagstimRapid 2; Figure-of-8 coil; 70-mm diameter	Contralateral cerebellum	CRB-iTBS; 80%AMT	2 groups of CRB-iTBS stimulation with 5min intervals	—	1200	1 time/d, lasting 3wk	NO
Lin^[25]^ 2019	–	Bilateral-LE M1	LE M1-iTBS; 100%MMT	Bursts of 3 pulses at 35 Hz applied at a rate of 5 Hz, a 2-s stimulation time with 10-s intervals	400 s	1200	2 times/wk, a total of 5wk, a total of 10 times	NO

AMT = the Active Motor Threshold, CRB = cerebellum, LE M1 = the primary motor cortex innervating the lower limbs, MEP = motor evoked potentials, RMT = the Resting Motor Threshold.

#### 3.3.2. Interventions

Regarding the TBS mode for stroke of the included studies, the iTBS mode was applied in 7 studies,^[14,15,20–23,25]^ except in the study of Li et al,^[24]^ who utilized the cTBS mode. Among these studies, 6 studies^[15,20–23,25]^ used iTBS plus CRT in the intervention group compared with sham iTBS plus CRT in the control group to observe the treatment effect. Wang et al^[14]^ used iTBS combined with CRT and suspension exercises, while Li et al^[24]^ utilized iTBS in combination with low-frequency rTMS, CRT, and acupuncture. Of the 6 studies,^[14,15,21–24]^ the targeted stimulation area of TBS intervention was the cerebellum, with 5 studies^[14,15,21–23]^ focusing on the contralateral cerebellum and 1 study^[24]^ targeting the right side of cerebellum. However, in another 2 studies,^[20,25]^ the targeted region for iTBS was the primary motor cortex (M1) controlling the lower limb.

#### 3.3.3. Essential TBS parameters

The relevant treatment parameters of iTBS included the selection of the output intensity, which was set at 80% of the active motor threshold,^[14,15,22–24]^ or within the range of 70 to 80% of the resting motor threshold,^[20,21]^ or set at 100% of the midline motor threshold^[25]^; the total stimulation duration, ranging from 80 to 400 seconds^[14,20–22,24,25]^; and the burst stimulation, which primarily was set at bursts of 3 pulses at 35 or 50 Hz applied at a rate of 5 Hz for a 2 or 20 seconds stimulation time with 8 or 10 second intervals.^[14,20–22,24,25]^ Furthermore, the number of total pulses ranged from 600 to 1200.^[14,15,20–25]^ The treatment cycles typically involved sessions occurring once or twice a day, lasting for a period of 2 to 5 weeks.^[14,15,20–25]^

### 3.4. Results of meta-analysis

#### 3.4.1. Primary outcomes

##### 3.4.1.1. Low limb motor function (FMA-LE)

**Figure 3.**
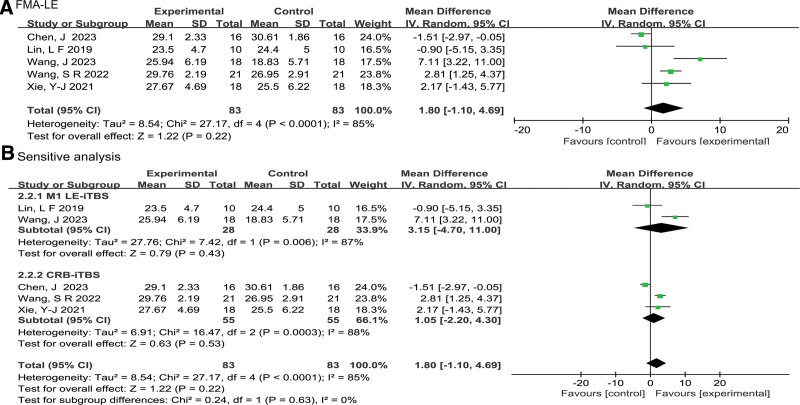
Effect of iTBS on recovery of lower limb motor function in stroke patients(A), and sensitivity analysis considering LE M1-iTBS and CRB-iTBS stimulation(B). FMA-LE = the Fugl-Meyer Assessment of Motor Recovery for lower extremity; LE M1-iTBS = iTBS stimulates the primary motor cortex innervating the lower limbs; CRB-iTBS = iTBS stimulates cerebellar.

Figure 3A shows a forest plot of the FMA-LE score. A total of 5 studies^[14,20–22,25]^ reported FMA-LE score and were included in this meta-analysis, with a total of 166 participants. A random-effects model was used to conduct a meta-analysis of FMA-LE scores due to the significant heterogeneity observed (*P* = .0003, *I^2^* = 85%). The result of the meta-analysis showed that iTBS intervention did not have a significant effect, but tend to increase FMA-LE scores in stroke patients as compared to the control group (MD = 1.80, 95% CI: −1.10 to 4.69, *Z* = 1.22, *P* = .22).

Considering the varied stimulating regions of TBS protocols used in the studies,^[14,20–22,25]^ we performed sensitivity analyses (LE M1-iTBS and CRB-iTBS). Figure 3B shows a detailed forest plot of the subgroup analysis for the FMA-LE score. The results of the LE M1-iTBS subgroup analysis indicated that applying iTBS to the primary motor cortex (M1), which innervates the lower limbs, did not significantly enhance FMA-LE scores (MD = 3.15, 95% CI: −4.70 to 11.00, *Z* = .79, *P* = .43). However, the combination of only 2 studies^[20,25]^ resulted in high heterogeneity (*P* = .006, *I^2^* = 87%). The statistical heterogeneity could be attributed to differences in the severity of the patients’ conditions and the duration of iTBS treatment. In the study conducted by Wang et al,^[20]^ patients in the subacute phase received a total of 18 sessions of LE M1-iTBS treatment over a period of 3 weeks, whereas in the study conducted by Lin et al,^[25]^ patients in the chronic phase got 10 sessions of LE M1-iTBS intervention over a period of 5 weeks.

For the CRB-iTBS subgroup, the application of iTBS to the contralateral cerebellum did not result in significant enhancement in FMA-LE scores (MD = 1.05, 95% CI: −2.20 to 4.30, *Z* = .63, *P* = .53). Nevertheless, 3 studies^[14,21,22]^ were included for combination, exhibiting a significant level of heterogeneity (*P* = .0003, *I^2^* = 88%). The variations in heterogeneity may be associated with the combination treatment regimen. Wang et al^[14]^ implemented CRB-iTBS treatment along with CRT and suspension exercise and CRT, while 2 other studies^[21,22]^ employed CRB-iTBS treatment in combination with only CRT.

##### 3.4.1.2. Balance function (BBS)

**Figure 4.**
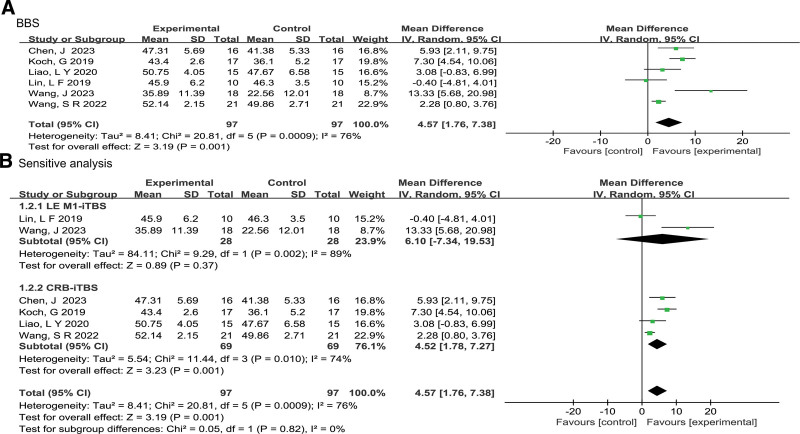
Effect of iTBS on balance recovery in stroke patients(A); Sensitivity analysis considering LE M1-iTBS and CRB-iTBS stimulation(B). BBS = Berg Balance Scale; LE M1 = the primary motor cortex innervating the lower limbs; CRB = cerebellum.

Figure 4A shows a forest plots of the BBS score. A total of 6 studies^[14,15,20,21,23,25]^ assessed the BBS scores and were included in this meta-analysis, with a total of 194 participants. The observed statistical heterogeneity was high(*P* = .0009, *I^2^* = 76%), therefore, we used a random-effects model to perform the meta-analysis. The results of the meta-analysis revealed that iTBS intervention had a significant effect to increase BBS scores in stroke patients as compared to the control group (MD = 4.57, 95% CI: 1.76 to 7.38, *Z* = 3.19, *P* = .001).

Given the significant heterogeneity, we performed sensitivity analyses based on differences in stimulating regions of TBS protocols (LE M1-iTBS and CRB-iTBS). Figure 4B displays a detailed forest plot of the subgroup analysis of the BBS scores. This results revealed that applying LE M1-iTBS did not significantly improve BBS scores (MD = 6.10, 95% CI: −7.34 to 19.53, *Z* = .89, *P* = .37). Nevertheless, the combined results of only 2 studies^[20,25]^ produced a high level of heterogeneity (*P* = .002, *I^2^* = 89%). We further found that differences in the severity of the patients’ conditions and the duration of iTBS treatment were correlated with the statistical heterogeneity. Wang et al^[20]^ included subacute patients with a total of 18 sessions of LE M1-iTBS interventions within 3 weeks, whereas Lin et al^[25]^ included chronic patients who received a total of 10 sessions of LE M1-iTBS interventions within 5 weeks.

For the CRB-iTBS subgroup, subgroup analysis showed that applying iTBS intervention to the contralateral cerebellum can significantly increase stroke patients’ BBS scores compared to the control group (MD = 4.52, 95% CI: 1.78 to 7.27, *Z* = 3.23, *P* = .001). The observed heterogeneity in the CRB-iTBS subgroup of the included 4 studies^[14,15,21,23]^ was moderate (*I^2^* = 74%). Therefore, we performed additional sensitivity analysis to identify the heterogeneity’s cause. After removing the Wang et al study,^[14]^ the findings of the sensitivity analysis revealed medium heterogeneity (*P* = .22, *I^2^* = 33%) in the CRB-iTBS subgroup. Wang et al^[14]^ received CRB-iTBS treatment in combination with CRT and suspension exercises, whereas the intervention group in the other studies^[15,20,23]^ involved CRB-iTBS treatment combined with CRT. Afterwards, the BBS scores (MD = 5.73, 95% CI: 3.31 to 8.16, *Z* = 4.63, *P* < .00001) in the CRB-iTBS intervention remained statistically significant as compared to the control group.

#### 3.4.2. Secondary outcomes

##### 3.4.2.1. Walking time (10MWT)

**Figure 5.**
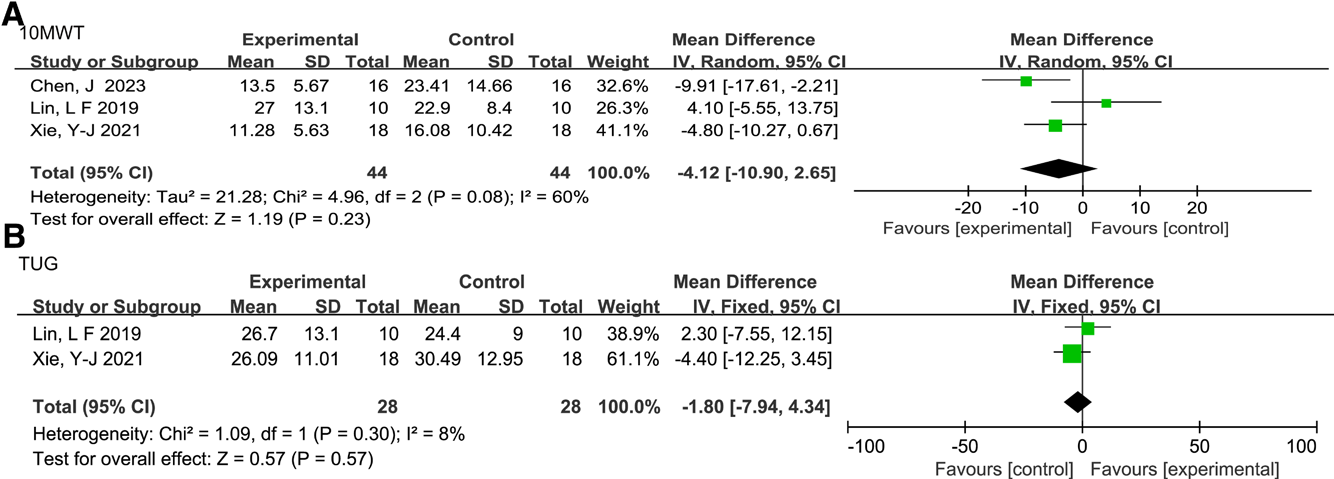
Effect of iTBS on walking time(A) and functional mobility(B) in stroke patients. 10MWT = 10-m walk test(s); TUG = Timed Up and Go test.

Figure 5A presents a detailed forest plot of the 10MWT(s) time. Three studies^[21,22,25]^ included 88 participants in the meta-analysis to investigate the effects of iTBS on 10MWT time. Considering the presence of medium heterogeneity (*P* = .08, *I^2^* = 60%), we used a random-effects model to calculate the MD. The result indicated that iTBS did not lead to a decrease in 10MWT time among stroke patients (MD = 4.12, 95% CI: −10.90 to 2.65, *Z* = 1.19, *P* = .23). Removing the study by Lin et al,^[25]^ which used the LE M1-iTBS intervention, resulted in low heterogeneity (*P* = .29, *I^2^* = 11%). However, the result suggested that the CRB-iTBS intervention significantly decreased the time to complete the 10MWT task (MD = −6.61, 95% CI: −11.39 to −1.82, *Z* = 2.70, *P* = .007).^[21,22]^

##### 3.4.2.2. Functional mobility(TUG)

Figure 5B provides a detailed forest plot of TUG time. Two studies^[22,25]^ included 56 participants in the meta-analysis to assess TUG time. We conducted a meta-analysis using a fixed-effects model due to the low heterogeneity (*P* = .30, *I^2^* = 8%). The results showed that iTBS did not improve the TUG time of stroke patients (MD = 1.80, 95% CI: −7.94 to 4.34, *Z* = .57, *P* = .57).

##### 3.4.2.3. Level of independence in daily living [(M)BI]

**Figure 6.**
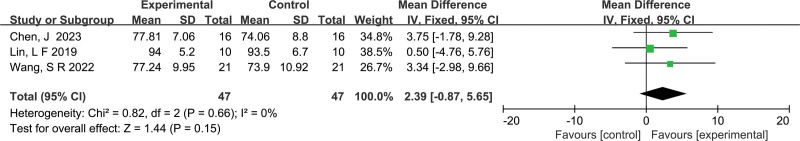
Effect of iTBS on level of independence in daily living[(M)BI] in stroke patients. (M)BI = (modified) Barthel Index.

Figure 6 shows a detailed forest plot of the (M)BI scores. Three studies^[14,21,25]^ reported the (M)BI scores and were included in this meta-analysis, with a total of 94 participants. Regarding the absence of heterogeneity (*P* = .66, *I^2^* = 0%), a fixed-effects model was utilized to calculate the MD. The meta-analysis results found that iTBS did not increase the (M)BI scores of stroke patients (MD = 2.39, 95% CI: -.87 to 5.65, *Z* = 1.44, *P* = .15) among stroke patients. After excluding the study that applied the LE M1-iTBS intervention,^[25]^ the CRB-iTBS intervention showed no statistically significant difference compared with the control group (MD = 3.57, 95% CI:-.59 to 7.73, *Z* = 1.68, *P* = .09).

##### 3.4.2.3. Corticospinal excitability (MEP latency)

**Figure 7.**
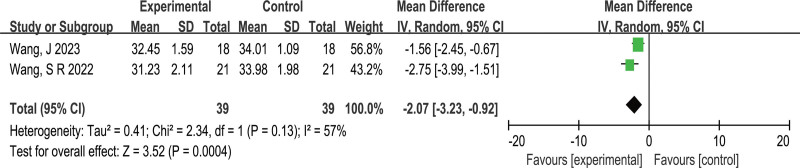
Effect of iTBS on Corticospinal Excitability in stroke patients. MEP latency = motor evoked potentials latency.

Figure 7 provides a detailed forest plot of MEP latency. Two studies^[14,20]^ reported the MEP latency and were included in this meta-analysis, with a total of 78 participants. We calculated the MD using a random-effects model due to the medium heterogeneity (*P* = .13, *I^2^* = 57%). The meta-analysis results showed that iTBS can markedly decrease MEP latency in stroke patients as compared to the control group (MD = −2.07, 95% CI: −3.23 to - .92, *Z* = 3.52, *P = *.0004).

#### 3.4.3. Adverse reactions

No relevant adverse reactions were reported in the literature included in the analysis, indicating a favorable safety profile of iTBS intervention for stroke patients with lower-limb motor dysfunction improvement and balance recovery.

## 4. Discussion

To our knowledge, this is the first systematic review and meta-analysis conducted to assess the clinical therapeutic efficacy of TBS in enhancing the restoration of lower-limb motor function and balance in stroke patients. From a total of 8 eligible studies^[14,15,20–25]^ of TBS and cTBS intervention with 290 participants that were included in the systematic review, 7 studies^[14,15,20–23,25]^ of iTBS intervention with 230 participants were included in meta-analysis. Presently, in contrast to the cTBS protocol, the iTBS protocol is more commonly used to improve lower-limb motor function and balance impairment in stroke patients. In the current meta-analysis, we found that TBS was effective in promoting balance recovery and reducing the latency of motor evoked potentials for patients with stroke, and the CRB-iTBS protocol may be more effective than the LE M1-iTBS protocol. However, despite there were no statistically significant differences in improving lower-limb motor function, walking performance, functional mobility, and activities of daily living as compared to the control group, TBS may offer important and promising therapy benefits.

### 4.1. Therapeutical effect of iTBS on lower-limb motor function

The present meta-analysis of 5 studies^[14,20–22,25]^ including 166 patients found that iTBS was not significantly effective in increasing FMA-LE scores. Additionally, no statistically significant differences were observed between the LE M1-iTBS and CRB-iTBS protocols for increasing FMA-LE scores. Nevertheless, iTBS shows promise as a potential treatment for improving lower extremity motor function. The rationale for using iTBS is a large amount of stimulation for the cerebral cortex through rapid pulse sequences, which may temporarily lower the excitability threshold in the affected M1 region. After stroke, the excitability of the affected hemisphere tends to decrease, whereas that of the contralateral hemisphere tends to increase, which results in the function of the affected hemisphere being inhibited by the contralateral hemisphere.^[26]^ However, studies have reported that the use of iTBS to target the LE M1 region in the affected cerebral hemisphere can directly increase its excitability^[27]^ and promote functional connectivity of the bilateral cerebral hemispheres.^[28]^

In addition, there are extensive neural network connections between the cerebellum and the cerebral cortex, especially in areas related to motor control, such as the M1 and supplementary motor area.^[29]^ It has been shown that iTBS targeting the contralateral cerebellum can indirectly increase the excitability of the motor cortex of the affected hemisphere.^[30]^ In the included studies, Wang et al^[14]^ of applying the contralateral CRB-iTBS and Wang et al^[20]^ of applying the affected LE M1-iTBS, both found that the MEP latency in the LE M1 area was significantly reduced. However, Xie et al^[22]^ and Liao et al^[23]^ reported no significant difference in the change in MEP amplitude between the iTBS intervention group and the control group when applying the contralateral CRB-iTBS. Furthermore, Koch et al^[15]^ demonstrated that the contralateral CRB-iTBS intervention resulted in an increase in the global mean field power (GMFP) of both the affected M1 area and the posterior parietal cortex compared to pretreatment, and the GMFP of the posterior parietal cortex significantly increased in the contralateral CRB-iTBS compared to the sham iTBS group. Therefore, these findings suggest that iTBS intervention may directly stimulate the affected M1 area to increase its excitability or activate the contralateral cerebellum to indirectly enhance the excitability of the motor cortex and parietal cortex. However, it is worth noting that there were significant differences in results between the iTBS group and the control group from different studies.

After enhancing the excitability of the motor cortex and parietal lobe, iTBS may offer potential neurophysiological support for the improvement of physical therapy in promoting lower-limb motor function. In 3 of 5 included studies assessing the CRB-iTBS protocol, it was observed that the combination of CRB-iTBS intervention with suspension exercise and CRT^[14]^ had a marked improvement in the FMA-LE score as compared to the CRB-iTBS intervention combined with CRT only.^[21,22]^ This difference in results may first be attributed to the impact of CRB-iTBS on the “cerebellar-cerebral loop,” which increased the effect of long-term potentiation in cortical excitability. Then, the application of suspension exercise enhanced the afferent proprioceptive information from the muscles, joints, and skin, making it easier to further activate “peripheral-central” neural loop efficiently, increasing sensory-motor integration and thus facilitating neuromuscular control. Finally, through the combined effect of activating the “central-peripheral-central” neural loop, the corticospinal excitability of the lower-limb motor was effectively promoted, thus the motor control of the low-limb was significantly improved.^[14]^ In addition, in 2 of 5 included studies, which assessed the LE M1-iTBS protocol, the enhancement of FMA-LE scores made by Wang et al^[20]^ was more notable than that achieved by Lin et al^[25]^ The main reason for this may be that Wang et al included stroke patients in the subacute phase, whereas Lin et al included patients in the chronic phase.

Therefore, under the premise that iTBS promotes cortical excitability of lower-limb motor, and considering that specific interventions tend to promote the activation of lower-limb control as a combined intervention, as well as performing iTBS intervention in the early stages of the disease, iTBS may be more likely to improve lower-limb motor function and produce more positive therapeutic effects in stroke patients.

### 4.2. Therapeutical effect of iTBS on balance recovery

Based on the findings of present meta-analysis including 6 studies^[14,15,20,21,23,25]^ with 194 patients, iTBS could markedly promote balance recovery in stroke patients. The potential effectiveness of iTBS in facilitating the restoration of balance may be attributable to its positive impact on cerebellar activity and the neural circuitry network that connects the cerebellum to the cerebrum. According to the current study, functional activity on 1 side of the cerebellar hemisphere is closely related to the activity and functional reorganization of neural networks in the motor cortex on the other side of the brain. In particular, the inhibitory influence is exerted by the cerebellum on the cerebral cortex, especially M1 area (known as cerebellar brain inhibition, CBI).^[15,29–33]^ Therefore, based on the theory of “cerebellar-cortical” functional connectivity, studies have found that CRB-iTBS could strongly activate the cerebellum, improve the CBI phenomenon, and further promote the improvement of individual balance,^[34,35]^ as well as the functional connection between the cerebellum and cerebral cortex.^[34–37]^

In the subgroup analysis for BBS score, the result demonstrated that the contralateral CRB-iTBS intervention significantly increased the BBS score, promoting balance recovery in stroke patients as compared to the control group. This result is consistent with the results of Wu et al’s meta-analysis,^[38]^ which revealed that noninvasive cerebellar stimulation enhanced the BBS score. This mechanism may involve iTBS stimulation to the cerebellum. On the 1 hand, CRB-iTBS stimulation can modulate the function of interneurons in the “cerebellar-cortical” circuit. iTBS reduced the excitability of the only efferent neurons in the cerebellum, the Purkinje cells,^[39]^ and thereby decreased the Purkinje cells inhibitory impact on the deep cerebellar nuclei,^[40]^ which ultimately promoted enhanced functional connectivity from the deep cerebellar nuclei to the motor cortex and parietal regions.^15,40–42^ On the other hand, CRB-iTBS stimulation can modulate the balance of glutamate and γ-aminobutyric acid(GABA) activities in the cerebral cortex through a “cerebellar-cortical” loop.^[43]^ This ultimately improved the imbalance between excitatory and inhibitory states in both cerebral hemispheres. Therefore, CRB-iTBS can activate the “cerebellar-cortical” loop to improve lower-limb motor control and visual-motor learning, and ultimately promote the recovery of balance in stroke patients.

Nevertheless, in contrast to the results of the CRB-iTBS intervention, LE M1-iTBS tended to promote balance recovery but was not statistically significant as compared to the control group. According to the “interhemispheric inhibition theory,”^[44]^ iTBS may tend to increase the excitability of the affected LE M1 area, improve the balance between excitatory and inhibitory states in bilateral cerebral hemispheres, and thus enhance balance recovery in the lower limbs. However, 2 studies^[20,25]^ reported that the effect of iTBS on balance recovery in the LE M1 area was not significant. This inconsistency observed may be due to the influence of intensity, duration, and treatment cycle of the iTBS intervention. Therefore, more RCTs assessing the effects of LE M1-iTBS protocols are required to validate these findings.

### 4.3. Therapeutical effect of iTBS on walking, functionanl mobility and independence in daily living

Stroke survivors generally suffer from significant impairments in walking and functional mobility, which are largely related to the decline in lower-limb motor control and balance function.^[45]^ The findings from this study indicated that iTBS resulted in a decrease in the time for the 10 MWT and TUG tests, as well as some improvement in walking performance. However, no statistically significant differences were observed when compared with the control group. The absence of substantial improvement might be related to various factors, including the extent of lower-limb motor function, degree of balance impairment, and a combination of interventions, treatment intensity, and duration. Due to the limited number of included studies, we should be prudent in interpreting these findings. Nevertheless, due to the advantages of iTBS, such as short-term stimulation and long-term potentiation, as well as its ability to significantly improve patients’ balance function, iTBS may be considered as a promising measure for enhancing walking and functional mobility. Therefore, we recommend that future studies include a greater number of RCTs to assess the effectiveness of iTBS in improving walking performance.

Studies have demonstrated a considerable association between walking performance, functional mobility, and independence in daily living.^[46,47]^ Our meta-analysis, which included 3 studies,^[14,20,25]^ revealed that the M(BI) score failed to exhibit a statistically significant improvement following the iTBS intervention as compared to the control group. This can be attributed to the fact that both the iTBS group and the control group received the same CRTs, and the upward trend in (M)BI scores may mainly be influenced by the efficacy of CRTs. Additionally, this may also be associated with the inadequate enhancement of lower-limb motor function and the restricted effectiveness of walking performance after iTBS treatment. Moreover, the results of the subgroup analysis demonstrated that the CRB-iTBS treatment during the subacute phase, with a duration of 3 to 4 weeks and 16 to 20 sessions,^[14,21]^ was more effective in enhancing independence in daily living compared to the LE M1-iTBS treatment during the chronic phase, which lasted for 5 weeks and comprised 10 sessions.^[25]^ The findings imply that subacute patients are more likely to enhance their degree of independence and that the iTBS intervention proves to be more effective within a shorter yet concentrated time. Therefore, when designing iTBS treatment plans, it is crucial to consider variables such as disease progression, sessions of iTBS stimulation, duration of treatment, and targeted site.

### 4.4. Research Limitations

Although this review reports some novel findings, we recognize several limitations. First, The significant heterogeneity in results may be related to differences in disease progression, level of functional impairment, stimulation region for iTBS, and the small sample size. Second, the heterogeneity was increased as a result of unclear allocation concealment, absence of blinding for both researchers and subjects, or the presence of open-label trials in certain studies. Finally, TBS is a new paradigm of rTMS, while iTBS is also a novel research direction for noninvasive brain stimulation techniques in stroke patients; as a result, there is a lack of high-quality RCTs available for inclusion in this study. Furthermore, due to the lack of a sufficient number of studies, publication bias was not assessed.

## 5. Conclusion

Based on the findings of the present review and meta-analysis, TBS, which regulates the excitability of the corticospinal pathway that controls lower-limb muscle movements, is a promising method to improve lower-limb dysfunction and promote balance recovery after a stroke. TBS combined with CRTs has a better effect on the recovery of balance function in patients with strokes than CRTs. The CRB-iTBS protocol may be more effective than the LE M1-iTBS protocol in terms of improving lower-limb balance recovery and walking performance. Meanwhile, TBS may be promising effects on lower-limb motor function, walking performance, and independent living. Nevertheless, in order to enhance the practical therapeutic effectiveness in stroke patients, future studies may need to consider integrating TBS with targeted walking training and physical therapies to facilitate the improvement of lower extremity motor function and balance recovery. However, owing to the significant heterogeneity present, future studies will need to include larger sample sizes and conduct rigorous, high-quality research to validate the exact therapeutic effects and clinical benefits of TBS in enhancing lower-limb motor function and balance recovery after strokes.

## Author contributions

**Conceptualization:** Kang Chen, He Zhuang.

**Data curation:** Kang Chen.

**Formal analysis:** Kang Chen.

**Funding acquisition:** He Zhuang.

**Investigation:** Kang Chen, Meixia Sun.

**Methodology:** Kang Chen, He Zhuang.

**Project administration:** Kang Chen, Meixia Sun.

**Resources:** Kang Chen.

**Visualization:** Meixia Sun, Kang Chen.

**Validation:** He Zhuang.

**Writing – original draft:** Kang Chen.
